# Household storage and disposal of unused and expired medicines in Dessie, Ethiopia: a cross-sectional study

**DOI:** 10.3389/fpubh.2024.1422304

**Published:** 2024-10-24

**Authors:** Alebachew Yimer, Getachew Moges, Mesfin Haile Kahissay

**Affiliations:** ^1^Department of Pharmacy, College of Medicine and Health Sciences, Wollo University, Dessie, Ethiopia; ^2^Department of Pharmaceutics and Social Pharmacy, School of Pharmacy, College of Health Sciences, Addis Ababa University, Addis Ababa, Ethiopia

**Keywords:** leftover medicines, outdated medicines, personal medication storage, medication disposal, Dessie, Ethiopia

## Abstract

**Introduction:**

Households frequently store unwanted, unused, or expired medicines in their homes indefinitely or discard them through general municipal waste bins, sinks, or flush them into their toilets. Disposing unused or expired medicines through these unauthorized channels can affect the environment and lives of individuals. This study assessed the household storage and disposal practices of unused and expired medicines in the Dessie City Administration in northeast Ethiopia.

**Methods:**

A community-based cross-sectional study was conducted using a semi-structured interview. The data were collected during November 1–30, 2019. A Multistage sampling technique was employed to recruit participants. The data were analyzed using SPSS version 23.

**Results:**

The study found that 62.4% of households stored unused or expired medicines, with analgesics, antibiotics, and cold and flu drugs being the most commonly used. The primary reasons for storing these medicines included future use, discontinuation of medication, and sharing with others, if needed. Most people preferred to dispose of these medicines by trashing them in household garbage, while some disposed of them in their original form and a few diluted them with water before disposal. Multivariate logistic regression analysis identified key factors associated with medicine storage: housewives and government employees were less likely to store unused medicines compared to students and daily workers, and obtaining medicines from private dispensaries was linked to reduced storage. Additionally, households that received advice from physicians were less inclined to store unused medicines.

**Conclusion:**

This revealed the widespread storage of unused medicines, primarily analgesics and antibiotics. Improper disposal practices are common and emphasize the need for public education. Employment status, source of medicines, and healthcare advice influenced storage behaviors. Healthcare providers play a vital role in improving medication management and reducing waste.

## Introduction

The utilization of prescription and over-the-counter (OTC) medications has seen a remarkable upswing on a global scale. However, several factors contribute to patients not using all the prescribed medications, including noncompliance, medication expiration, improved medical conditions, excessively large medication supplies, patient demise, and alterations in prescriptions due to side effects or a lack of therapeutic efficacy. Doubts about the necessity of medications, fear of adverse reactions, and forgetfulness can also lead to self-discontinuation. These factors collectively result in the accumulation of unused or expired medications within households and contribute to medication wastage (1–3). Shockingly, more than half of all medications are inappropriately prescribed and dispensed, leading to unnecessary storage and posing environmental threats (1, 3).

Pharmaceuticals are used not only for human medicine but also in veterinary medicine, and this also has environmental impacts (4). Household pharmaceuticals can find their way into the environment through various routes, such as excretion after ingestion, injection, or infusion, removal of topical medications during bathing, and the disposal of unwanted or leftover pharmaceuticals (5). Households often store unwanted, unused, or expired medications indefinitely or dispose of them improperly through general municipal waste bins, sinks, or toilets. Disposing of unused or expired medications through these unauthorized channels can lead to environmental pollution, impacting both humans and animals, as well as contaminating soil and water supplies (6).

The storage of unused or expired medicines in households carries several risks, including drug resistance, diversion for initial use, accidental ingestion by children, and overdose, especially during emergencies. Continuous release of medicines into the environment, even at trace levels, may result in long-term exposure, posing chronic threats to humans and wildlife (7). For example, the presence of antidepressants in water bodies has been shown to alter the behavior of fish, making them less cautious and more vulnerable to predators. Additionally, exposure to hormones from contraceptive pills can cause reproductive issues in aquatic species, such as the feminization of male fish (8). Prolonged exposure to pharmaceuticals in the environment, especially for vulnerable populations such as pregnant women, newborns, and children, can have hazardous effects (8, 9). The presence of antibiotics in water bodies contributes to the emergence of antibiotic resistance, potentially leading to gene abnormalities in both humans and aquatic life (10).

Therefore, the risks associated with the household storage of unused and expired medicines have garnered the attention of policymakers, healthcare professionals, pharmaceutical companies, and communities worldwide (11). Studies conducted in various African countries have revealed unsafe disposal practices for unused and expired medicines, characterized by unregulated, illegal, and indiscriminate disposal (12). In Ethiopia, there are currently no national policies governing the disposal of unused and expired medicines. Encouraging safe and appropriate disposal practices within communities and drawing the government’s attention to this issue are of paramount importance. The extent and nature of the problem related to unused, expired, and unwanted medicines have not been comprehensively studied in Ethiopia. This study aims to assess household storage and disposal practices for unused and expired medicines in Dessie City Administration, North East Ethiopia.

## Methods

### Study area and period

The study was conducted in Dessie City Administration, Amhara Regional State, Ethiopia, from November 1 to 30, 2019. Dessie City Administration is located 401 km from the capital city of Ethiopia, Addis Ababa. Administratively, it comprises five sub-cities, totaling 18 urban kebeles and eight rural kebeles, with a population of 421,930 people. Among these, 383,011 reside in urban areas and 38,919 in rural areas, according to the Dessie City Administration Health Office’s population profile for 2019. Dessie boasts infrastructure that includes one governmental university, one governmental Health Science College, and five private Health Science Colleges. Additionally, there are two governmental and three private hospitals, eight governmental health centers, two medical and surgical specialty centers, eight specialty clinics, 31 medium clinics, and 11 private primary clinics. Alongside the Ethiopian Pharmaceuticals Supply Agency (EPSA), there are 20 pharmaceutical wholesalers, 20 private pharmacies, three model community pharmacies, one Red Cross pharmacy, and 26 private drug shops.

### Study design and population

A community-based cross-sectional study was conducted among households in Dessie City. The source population included all households in Dessie City. Heads of households or any adult household member aged 18 years and older, who were well-informed about the family’s medication-taking behavior, available during the data collection period, and free from mental illness, were eligible for inclusion.

### Sample size determination and sampling procedure

The sample size was determined using the single population proportion formula. Based on a finding by Wondimu et al. the proportion of households stocking unused or expired medicines was 19% (13). With a 5% tolerable sampling error at a 95% confidence level, a 10% contingency for non-response, and a design effect of 2, the final sample size was calculated to be 521 households.

A two-stage sampling procedure was employed. In the first stage, Dessie City was divided into 18 urban and eight rural kebeles. Two urban and one rural kebele were proportionally allocated and selected using a simple random sampling technique. In the second stage, the calculated number of households was recruited for the study using systematic random sampling and proportional allocation techniques. If a household member did not meet the inclusion criteria, the immediate next household was selected. For qualitative data collection, seven key informants were purposively selected to engage with individuals knowledgeable about the study topic.

### Data collection and management

Data were collected by five trained senior pharmacists using structured interviewer-administered questionnaires and direct observation. The investigator supervised the data collectors throughout the data collection period to ensure data quality. A pretest of the questionnaire was conducted on 26 households not included in the actual study. All completed questionnaires were carefully examined for completeness and consistency during data management, storage, and analysis.

The questionnaire comprised two sections. The first section gathered general demographic information, while the second section focused on the storage of unused or expired medicines, reasons for such storage, methods of disposal, and awareness of disposal practices for these medicines.

### Data entry and processing

All collected questionnaires underwent thorough sorting and examination for quality and accuracy before statistical analysis. Data were checked for completeness, and coding was performed either before or after the collection of data. The data were then entered into Epi-Data software version 4.6.0 and later exported to SPSS software version 20 for analysis. Descriptive statistics, binary logistic regression, and multiple logistic regression analysis were conducted. Raw data were coded and recoded following the software’s operating procedures. In bivariate analysis, factors with a *p*-value less than or equal to 0.25 were retained and entered into multivariable logistic regression analysis. Factors with a *p*-value less than 0.05 were considered important predictors of the outcome variable.

### Ethics approval and consent to participate

Ethical clearance was obtained from the College of Medicine and Health Sciences Ethical committee, Wollo University (CMHS411/13/11). Letter of cooperation was written to Zonal health department. Permission was obtained from Woreda health office before starting data collection. The participants were informed about the purpose of the study and verbal consent was obtained from each participant The College of Medicine and Health Science Ethical Committee, Wollo University also approved the informed verbal consent process and to ensure patient confidentiality, participants were not identified by names or other personal identifiers. The study was conducted in accordance with the Declaration of Helsinki.

### Reflexivity issues: AYA status as an insider

The principal investigator approached this work as a native Amharic speaker and resident of the study communities. As a member of the Amhara community and an expert in pharmacy, the investigator utilized existing networks and contacts within health institutions in both the governmental and private pharmaceutical sectors to gain access to various individuals. The researcher remained mindful of how the data collection process might influence personal perceptions and the responses of others. To mitigate the potential perception of being an expert pharmacist, the researcher employed semi-structured and open-ended questions and engaged in informal conversations with participants on topics they themselves raised.

## Results

### Socio-demographic characteristics of participants

A total of 503 households responded to the interviewer administered questionnaire giving a response rate of 96.5%. The majority 319 (63.4%) of the respondents were females. The mean age of the respondents was 34.86 years. Nearly one third, 158 (29.4%) of the respondents were housewives. Regarding educational status of the household head, 224 (44.5%) attained college and above levels. The majority of the respondents 385 (76.6%) were married. There was no health professional in more than three-fourth (79.5%) of the households (Table 1).

**Table 1:** Sociodemographic characteristics of respondents in Dessie City; North East Ethiopia (*N* = 503; November 2019).

Variable	Frequency	Percentage
Gender		
Male	184	36.6
Female	319	63.4
Age (years)		
18–30	228	45.3
31–50	220	43.7
>50	55	10.9
Marital status		
Married	385	76.6
Single	88	17.4
Widow/widower	16	3.2
Divorced	14	2.8
Religion		
Orthodox Christian	256	50.9
Muslim	200	39.8
Protestant	35	7.0
Catholic	11	2.2
Household head’s education level		
Illiterate	25	5.0
Can read and write	52	10.3
Primary education	53	10.5
Secondary education	149	29.6
College and above	224	44.5
Occupation		
Government employee	99	19.7
Private employee	59	11.7
Merchant	60	12.1
Housewife	148	29.4
unemployed	52	11.9
Others^*^	85	16.9
Family size		
≤5	396	78.7
>5	107	21.3
Presence of health professional in the family		
Yes	103	20.5
No	400	79.5

* Students, daily workers, waiters, farmer, retired.

### Household storage of unused and expired medicines

Three hundred fourteen (62.4%) of the households studied stored unused or expired medicines at home. The most frequently reported reasons for having unused and expired medicines stored at homes were for future use 284 (90%), discontinuation of medication 219 (69.7%), and to give others if needed 115 (36.6%). Of those who had unused and expired medicines because of discontinuation of medication, the majority 175 (79.9%) discontinued because of improvement of medical conditions and more than one-third 77 (35.2%) discontinued because of change of medication (Table 2).

**Table 2:** Household storage of unused and expired medicines among households in South Wollo zone, North East Ethiopia, November 1–30, 2019 (503).

Questions	Frequency	Percentage
1. Do you or anyone in your household ever had unused medicines at your home?		
Yes	314	62.4
No	189	37.6
1. If yes, what was the reason for keeping unused medicines? (*n* = 314) ^*^ For future use	284	90
To give others if needed	115	36.6
Do not know how to discard	19	6
Do not want to waste them	38	12
Discontinuation of medication	219	69.7
Other^a^	4	1.2
2. If you or anyone in your household had unused medicines because of discontinuation of medication, why did you discontinue your medication? (*n* = 219) ^*^		
Intolerable side effect	25	11.4
Unclear labels or instructions	8	3.6
Medical conditions improved or resolved	175	79.9
Change of medication	77	35.2
Not sure why medication was prescribed	16	7.3
Difficulty of following medication instructions	10	4.6
Other^b^	2	0.9

* A single household may store more than one drug and multiple responses are possible. Therefore, percentages are greater than 100.

a Death of patients, to show for pharmacists and physicians for refill or next visit.

b Non adherence, forgetfulness.

### Categories of unused and expired medicines stored at homes

The most common categories of medicines stored at homes were analgesics followed by cold and flu remedies and antibiotics, stored by 162 (32.2%), 81 (16%), and 81 (16%) of the households, respectively, while the least common categories of medicines stored at homes were anti-epileptics stored by 6 (1.2%) of the households (Table 3).

**Table 3:** Categories of unused and expired medicines stored at homes in households of South Wollo zone, North East Ethiopia, November 1–30, 2019 (*n* = 503).

Category of medicines	Frequency	Percentage
Analgesics	162	32.2
Cold and flu remedies	81	16
Antibiotics	81	16
Anti-allergies	54	10.7
Antacids	51	10
Anti-hypertensive drugs	36	7.15
Anti-diabetics	29	5.8
Anti-diarrheal drugs	28	5.6
Anti-vomiting	26	5.2
Anti-epileptics	6	1.2
Other^*^	51	10

* Anti- fungals, CNS drugs, ART, vitamins & minerals, COC, eye drops, steroids, GI drugs.

### Categories of diseases the unused and expired medicines are used for

The unused or expired medicines stored in the households were acquired for managing different types of diseases of the households which stored unused or expired medicines, three-fourth (75%) reported that their stocked medicines were for the management of seasonal diseases, 192 (61%) reported that the stocked medicines were for the management of acute diseases and 75 (24%) reported that the stocked medicines were meant for the management of chronic conditions.

### Disposal practice of unused and expired medicines

The most chosen disposal practice of unused or expire medicines were trashing in household garbage 328 (65.2%) followed by flushing down in to the toilet or the sink 185 (36.9%), and burning 172 (34.2%). More than half, 268 (53.2%) of the households disposed unused or expired medicines as they are and one-tenth 46 (9.1%) of them disposed by diluting with water, while one-third 142 (28.2%) did not know how to dispose unused and expired medicines (Table 4).

**Table 4:** Disposal practice of unused and expired medicines among households in Dessie city administration, North East Ethiopia, November 1–30, 2019 (*n* = 503).

Questions	Frequency	Percentage	
1. What do you do with unused or expired medicines?^*^ Thrash in household garbage	328	65.2	
Flush down in to the toilet/sink	185	36.8	
Throw away to the environment	32	6.4	
Bury in the ground		117	23.3
Keep at home until expired	61	12	
Burn	172	34	
Return back to pharmacy	3	0.6	
Other^a^	13	2.6	
2. How do you dispose unused or expired medicines? Crash before discarding	47	9.3	
Dilute with water	46	9.1	
As it is	268	53.2	
I do not know what to do	142	28.2	

* Multiple responses are possible. Therefore, percentages are greater than 100.

a Return back to pharmacy, give to friends or relatives, keep them in home.

### Perceptions about disposal of unused and expired medicines

The majority, (92.8%) of the respondents perceived that unused and expired medicines present potential risks at home. More than half, (58.6%) of the respondents perceived the negative impact of throwing medicines in the garbage or flushing down them in to the toilet or the sink. Regarding information on safe disposal of unused or expired medicines, the majority (87.3%) perceived that there was lack of adequate information on safe disposal of unused and expired medicines. The majority, 358 (71.2%) of the participants agreed on mandatory take-back programs for the disposal of unused and expired medicines (Table 5).

**Table 5:** Perceptions about disposal of unused and expired medicines among households in Dessie city administration, North East Ethiopia, November 1–30, 2019 (*n* = 503).

Statements	SD_n(%)_	DA_n(%)_	N_n(%)_	A_n(%)_	SA_n(%)_
1. Unused and expired medicines present potential risks at home.	6 (1.2)	11 (2.2)	19 (3.8)	228 (45.3)	239 (47.5)
2. Throwing medications in the garbage or flushing down them in to in to the toilet or the sink has a detrimental effect on the environment.	50 (1)	70 (13.9)	88 (17.5)	150 (29.8)	145 (28.8)
3. Children are more vulnerable to the risks of associated with unused or expired medicines at home.	4 (0.8)	4 (0.8)	90 (1.8)	177 (35.2)	309 (61.4)
4. There is lack of adequate information on safe disposal of unused or expired household medicines.	8 (1.6)	23 (4.6)	32 (6.4)	277 (55.1)	162 (32.2)
5. Healthcare professionals do provide advice on safe disposal of unused or expired household medicines.	4 (0.8)	5 (1.0)	21 (4.2)	247 (49.1)	226 (44.9)
6. Take-back programs of unused or expired medicines should be mandatory.	10 (2)	62 (12.3)	73 (14.5)	196 (39)	162 (32.2)

SD, strongly disagree; DA, disagree; N, neutral; A, agree; SA, strongly disagree.

### Logistic regression on predictors of storage of unused medicine

In the multivariate logistic regression analysis, certain occupational statuses were found to be significantly associated with the storage of unused medicines in households. Specifically, individuals working as housewives and government employees had a notably lower likelihood of storing unused medicines compared to those in other occupations, such as students or daily workers, within their households (AOR = 0.377, 95% CI: 0.166–0.858, and AOR = 0.396, 95% CI: 0.175–0.894, respectively). Moreover, households that sourced their medicines from private dispensaries were significantly less likely to store unused medicines compared to those that did not acquire medicines from such dispensaries (AOR = 0.480, 95% CI: 0.296–0.777). Additionally, households receiving advice from physicians had lower odds of storing unused medicines than those not advised on medicine storage (AOR = 0.654, 95% CI: 0.437–0.978).

Furthermore, the types of diseases prevalent in households played a significant role in medicine storage practices. Specifically, households dealing with chronic and acute diseases were found to have a significantly lower chance of storing drugs, with odds ratios of 0.168 (95% CI: 0.083–0.339) and 0.646 (95% CI: 0.428–0.975), respectively, compared to households with seasonal diseases, all with a *p*-value less than 0.05 (Table 6). The model’s goodness-of-fit was assessed using the Hosmer and Lemeshow test, resulting in a non-significant *p*-value (>0.05) of 0.956, indicating that the model effectively fits the data.

**Table 6:** Multi-variate logistic regression test for predictors of presence of medicines in households, Dessie, North East Ethiopia (*N* = 503, November 2019).

Variable	Medicines in household		*p* value	AOR (95% CI)
	Yes (%)	No (%)		
Educational status				
Not read and write	14 (56.0)	11 (44.0)	0.692	1.229 (0.444–3.398)
Read and write	29 (58.8)	23 (44.2)	0.654	0.847 (0.411–1.749)
Primary education	25 (47.2)	28 (52.8)	0.769	1.112 (0.547–2.264)
Secondary education	56 (37.6)	93 (62.4)	0.154	1.467 (0.870–2.473)^**^
College/university education	111 (49.6)	113 (50.4)	0.526	1
Health related professional in household				
Yes	60 (58.3)	43 (41.7)	0.078	0.630 (0.377–1.054)^**^
No	175 (43.8)	225 (56.2)		1
Occupation				
Housewife	75 (50.7)	73 (49.3)	0.020	0.377 (0.166–0.858)^*^
Govt. employee	51 (51.5)	48 (48.5)	0.026	0.396 (0.175–0.894)^*^
Private employee	24 (40.7)	35 (59.3)	0.305	0.628 (0.259–1.526)
Retired	18 (64.3)	10 (35.7)	0.166	0.433 (0.133–1.416)^**^
No work	22 (42.3)	30 (57.7)	0.272	0.600 (0.242–1.491)
Merchant	27 (44.3)	34 (55.7)	0.106	0.471 (0.189–1.173)
Farming	5 (45.5)	6 (54.5)	0.349	0.472 (0.098–2.274)
Others^***^	13 (28.9)	32 (71.1)	0.343	1
Types of disease to take medicine				
Chronic	61 (81.3)	14 (18.7)	0.0001	0.168 (0.083–0.339)^*^
Acute	90 (46.9)	102 (53.1)	0.038	0.646 (0.428–0.975)^*^
Seasonal	83 (35.3)	152 (64.7)	0.0001	1
Private dispensaries				
Yes	195 (51.0)	187 (49.0)	0.003	0.480 (0.296–0.777)^*^
No	40 (33.1)	81 (66.9)		1
Physicians				
Yes	121 (57.6)	89 (42.4)	0.039	0.654 (0.437–0.978)^*^
No	114 (38.9)	179 (61.1)		1
Total family category				
1–5	179 (45.2)	257 (54.8)	0.923	0.976 (0.590–1.613)
6–10	56 (52.3)	51 (47.7)		1

^*^Significance association, ^**^Non significance association, ^***^Students, daily workers.

## Discussion

This study aimed to assess household storage and disposal practices of unused and expired medicines in Dessie City, North East Ethiopia. The findings revealed that a majority of households, specifically 314 (62.4%), stored unused or expired medicines. This rate was higher than in several other studies conducted in different regions, such as Agew Awi zone, North West Ethiopia (13.8%) (14), Mekelle, North Ethiopia (41.4%) (15), Kenya (51.83%), and Turkey (28.0%) (16). However, it was lower than reported rates in Johannesburg (76.24%) (17), Kabul (95%) (18), and Ireland (88%) (19). These variations may be attributed to disparities in access to pharmacies, differences in self-medication practices, the high cost of medicines, a lack of awareness about proper disposal methods, and the possibility of future need for these medicines. Similar findings were documented in studies conducted in Harar, East Ethiopia (66.6%) (20), Nigeria (65.8%) (21), and New Zealand (62%) (22).

The primary reason reported for keeping unused or expired medicines in households was for future use (90%), followed by discontinuation of medication (69.7%) and keeping them to share with others if needed (36.6%). This aligns with a study in Ireland where the most common reason for retaining unused medicines was “in case they are needed later” (68%) (19). A study in India found that the most frequent reasons for keeping unused medicines were discontinuation due to recovery from illnesses (61.4%) and the intention to reuse the medicines in the near future (57.6%) (23). In this study, discontinuation of medication was the second most frequently reported reason for having unused or expired medicines at home. This pattern suggests a lack of awareness among households in Dessie city regarding the importance of completing the full course of therapy. Notably, more than one-third of respondents in the present study kept unused medicines at home to share with friends or family members. This practice poses several risks, including polypharmacy, multiple chronic comorbidities, and unsupervised drug use, highlighting the need for interventions to address these issues. Increased storage of unused or expired medicines in households also raises concerns about accidental childhood poisonings and environmental contamination.

The types of medicines most commonly stored at homes in this study were analgesics (32.2%), followed by antibiotics and cold and flu remedies, each stored by 16% of households, respectively. The higher prevalence of analgesics may be attributed to their wide use in treating various illnesses associated with pain. However, the percentage of households storing antibiotics was higher than in some other studies, such as in the USA (6.7%) (24) and India (8.5%). This indicates that many of these medicines were discontinued before completing the full course of treatment or were stored for future use, potentially contributing to antimicrobial resistance. The impacts of microbial resistance and its challenges are profound. The improper disposal of antibiotics contributes to the development of antibiotic-resistant bacteria, which can spread through the environment and pose significant public health risks. This resistance makes infections harder to treat, leading to longer hospital stays, higher medical costs, and increased mortality (6).

Regarding disposal practices, the majority of participants preferred trashing unused or expired medicines in household garbage (65.2%), followed by flushing them into the toilet or sink (36.8%). This prevalence was higher than in studies conducted in Harar (50%) (20), India (12%) (25), Saudi Arabia (48.1%) (26), and the USA (54%) (27). These findings may be due to a lack of awareness about proper disposal methods, the unavailability of nearby appropriate disposal sites, the convenience of trashing medicines in household garbage, and low awareness of the potential environmental harm resulting from improper disposal. Trashing medicines in household garbage poses risks of accidental poisonings in humans and pets and environmental contamination. For cases of medications being disposed of in sinks and toilets, it is important to note the wastewater treatment methods used in Dessie. Dessie relies on conventional wastewater treatment processes, which are generally ineffective at completely removing pharmaceutical compounds. As a result, these substances can persist in treated water, leading to environmental contamination (6, 28). Therefore, healthcare professionals should educate consumers about alternative disposal methods, such as mixing medicine with unappealing substances before trashing them in household garbage. Similar findings were reported in studies conducted in Adigrat (5)(63%) and the UK (63%) (29).

A noteworthy finding was that more than half of the participants (53.2%) preferred to discard unused or expired medicines in their original package and dosage form, contrary to recommendations by the American Food and Drug Administration (FDA) and the White House Office of National Drug Control Policy. These agencies suggest removing prescription medicines from their original containers, mixing them with undesirable substances, and sealing them in a plastic bag before disposal. Therefore, healthcare professionals in areas like Dessie City, where established drug take-back systems are unavailable, should educate the public about these alternatives. Dessie currently has no reverse logistics for expired or unused medications, there is a pressing need for regulations for this purpose. Countries such as Germany and Sweden have implemented laws mandating the proper return and disposal of unused pharmaceuticals to prevent environmental contamination and misuse (30).

Despite nearly three-quarters of participants expressing support for mandatory take-back programs for the disposal of unused and expired medicines, only a small percentage (0.6%) returned such medicines to pharmacies. This rate was much lower than in studies conducted in Nigeria (12.3%) (45), Turkey (34.0%) (16), Sweden (43%) (31), and the UK (22%) (29). The disparity may be due to the absence of established drug take-back systems in Ethiopia. Another pertinent factor to discuss is whether pharmaceuticals are included in federal environmental regulations that mandate their detection in the environment or in water intended for public supply. Currently, Ethiopia does not have specific regulations mandating the detection of pharmaceuticals in the environment or in water supplies. Implementing such regulations, as seen in countries like the USA and the EU, would be crucial in mitigating the environmental and public health impacts of pharmaceutical contaminants (32).

Although a high proportion of participants (87%) understood the negative impact of improper disposal of unused and expired medicines on the environment, two-thirds of them preferred to dispose of these medicines by trashing them in household garbage, which is detrimental to public well-being and the environment. This highlights the need for greater public education to raise awareness about proper disposal methods, a responsibility that should be shouldered by healthcare professionals, mass media, and environmental agencies. Regarding intoxications there is a lack of specific data on medication-related intoxications in Dessie. Thus, the establishment of a monitoring system for such incidents would provide valuable data for addressing this public health concern.

The results of the multivariate logistic regression analysis shed light on several factors associated with the storage of unused medicines in households, offering valuable insights in line with existing literature. The finding that housewives and government employees are less likely to store unused medicines compared to students and daily workers aligns with previous studies. Asmamaw et al. (33) conducted a study in a different setting and found that individuals with full-time employment tend to exhibit better medication management practices, potentially attributed to the structured daily routines and access to healthcare information often associated with formal employment (33).

Furthermore, the result indicating that households obtaining medicines from private dispensaries are less likely to store unused medications is consistent with the findings of Teni et al. (34). Patel and colleagues noted that individuals who receive medicines from private healthcare providers are more likely to follow proper medication management practices, including avoiding the accumulation of unused drugs. This suggests that private healthcare providers may be more diligent in prescribing the appropriate quantity of medications and providing clear instructions on their use (34).

Moreover, the result highlighting that households receiving advice from physicians are less likely to store unused medicines resonates with the study by Seehusen and Edwards (35). Dean and John found that healthcare professionals’ guidance plays a pivotal role in ensuring proper medication adherence and disposal practices. Physicians, in particular, are well-positioned to educate patients on the importance of adhering to prescribed regimens and safely disposing of unused medications (35).

The association between the types of diseases and the likelihood of storing unused medicines is consistent with the findings of Jassim (36). Their research revealed that households managing chronic and acute conditions tend to have a lower likelihood of accumulating unused medications. This observation may be attributed to the more regular and long-term use of medications in these households, leading to fewer surplus drugs (36).

In summary, these findings underscore the significance of considering socioeconomic factors, healthcare provider influence, and the nature of medical conditions when addressing the issue of medication storage. Interventions aimed at promoting proper medication management should take into account these multifaceted influences on household behaviors. Furthermore, healthcare providers, especially physicians, should play an active role in patient education regarding medication adherence and safe disposal practices, ultimately contributing to improved public health outcomes and reduced medication waste. Further research is warranted to explore these dynamics in greater depth and across diverse populations.

## Practical implication

This study highlights the critical importance of promoting safe storage and appropriate disposal practices for unused and expired medicines. Healthcare professionals should actively educate patients about the potential risks associated with improper medication handling and disposal. Policymakers and regulatory authorities must consider enforcing stringent regulations governing the dispensing and purchase of medications and develop comprehensive programs for proper disposal. This could involve the establishment of take-back programs, community-wide awareness campaigns, and developing educational materials to raise awareness about this issue throughout society. Collaboration among various stakeholders is essential. By addressing these issues collectively, we can significantly enhance medication safety and mitigate potential environmental and public health risks associated with improper pharmaceutical handling and disposal. Further research is needed to explore these dynamics in greater depth and across diverse populations.

## Limitations

This study has several limitations. First, the data collected relied on self-reporting, which may introduce recall bias. Second, this study employed cross-sectional design, data were collected at a point in time. Third, the study did not assess the environmental impact of improper medication disposal. Further research could address these limitations and explore the environmental consequences of improper disposal practices.

## Conclusion

In conclusion, this study investigated the storage and disposal practices of unused and expired medicines in Dessie City, North East Ethiopia. The majority of households were found to store such medicines, with reasons ranging from future use to discontinuation of medication. The prevalence of storage was higher than in some other regions but lower than in certain international studies. Analgesics were the most commonly stored medicines, possibly due to their frequent use in managing various health issues. However, the high storage of antibiotics raised concerns about potential antimicrobial resistance. Improper disposal practices, such as trashing medicines in household garbage, were prevalent, highlighting the need for better public education on appropriate disposal methods. The multivariate logistic regression analysis revealed that factors like employment status, the source of medicines, and healthcare professional advice influenced storage behaviors. These findings stress the importance of considering various factors when addressing medication storage issues and emphasize the pivotal role of healthcare providers in patient education to improve medication management and reduce waste. Further research is necessary to explore these dynamics across diverse populations and in greater detail.

## Data Availability

The raw data supporting the conclusions of this article will be made available by the authors, without undue reservation.
